# Corrosion Layers on Archaeological Cast Iron from Nanhai I

**DOI:** 10.3390/ma15144980

**Published:** 2022-07-18

**Authors:** Minghao Jia, Pei Hu, Gang Hu

**Affiliations:** School of Archaeology and Museology, Peking University, Beijing 100871, China; 2001110770@stu.pku.edu.cn (M.J.); 1801110803@pku.edu.cn (P.H.)

**Keywords:** Nanhai I, archaeological iron, corrosion layers, conservation

## Abstract

Archaeological iron objects were excavated from the Nanhai I ship from the Southern Song Dynasty that sunk in the South China Sea. Most of these artifacts were severely corroded and fragmented. In order to understand their current corrosion state and guide their restoration and protection, optical microscopy, scanning electron microscopy, micro-laser Raman spectroscopy, infrared spectroscopy and X-ray diffraction were all selected for analysis. It was clear that the archaeological iron material was hypereutectic white iron with a carbon content of about 4.3–6.69%, and had experienced low-melt undercooling. There were many internal cracks formed by general corrosion that extended to the iron core, which tended to make the material unstable. At the interface between the iron and rust, there was a black dense layer enriched with chlorine, and a loose yellow outer layer. The dense layer was mainly composed of magnetite, akaganeite and maghemite, while the rust of the loose layer was composed of lepidocrocite, goethite, feroxyhite, maghemite and hematite. The major phases of all corrosion products were akaganeite and lepidocrocite. Numerous holes and cracks in the rust layer exhibited no barrier ability to the outside electrolyte, hence the iron core formed many redox electrochemical sites for general corrosion with the rust. Meanwhile, the dense rust located close to the iron core was broken locally by an enriched chlorine layer that was extremely detrimental to the stability of the archaeological iron. Using electrochemical impedance spectroscopy, it could be determined that the rust layers had no protective effect on the internal iron core under conditions of simulated seawater, and these rust layers even accelerated the corrosion. A mechanism for the rust growth as a result of laboratory testing was proposed to explain the entire corrosion process. In view of the desalination preservation treatment that had been applied for ten years, it was not recommended to maintain a single desalination operation. The archaeological rusted iron of the Nanhai I ship that was excavated from the marine environment should be properly stabilized and protected using corrosion inhibition and rust transformation for iron oxyhydroxides, since the rust structure and the internal iron core retain well together.

## 1. Introduction

The Nanhai I is a wooden merchant ship from the Southern Song Dynasty (1127–1279 AD), which was sunk in the South China Sea. It was unearthed in Guangdong province and is now a collection in the Maritime Silk Road Museum [1]. It conforms to the hull structure of a “Fu Ship (Fujian-style freighter)” from the typical types of Chinese ships. It is the oldest, largest and best-preserved shipwreck ever found for ancient international trade. The ship dates to the mid-13th century, as a result of the copper coins and china commodities that were unearthed from it [2]. The Song Dynasty was a period of rapid development in maritime trade. Developments in shipbuilding technology and navigation knowledge further expanded the scope of marine trade during this period, and communication with overseas became more frequent. Cargo from the Nanhai I reflects the trade situation during the Song Dynasty, filling gaps in the physical data of Sino-foreign maritime trade in academic research. The cargo salvaged from the shipwreck is of a wide variety and is on a large scale, retaining most of the products from that time. It provides us with new ideas for studying the maritime metals trade and navigation history from the Song Dynasty. Therefore, there are good reasons to conduct pre-conservation guided research on excavated archaeological artifacts, especially metal objects from the Nanhai I.

In ancient trade, metal products dominated the international market, and were important commodities of market circulation. Steel or iron can be used to manufacture weapons and labor tools. It was an indispensable military material that was closely related to the country’s politics, economy and military at that time [3]. Therefore, the study of metal artifacts is beneficial, since it reveals the influence of ancient China in the historical development of Asia and the world. A large number of daily necessities and commercial products which were made of iron or steel, such as pots, nails and long slabs, were excavated from the Nanhai I. Finding a method to stabilize the material under favorable conditions against further corrosion has been a serious problem. Further corrosion of the iron objects often occurs after extraction from their marine environment, and their exposure to a new environment tends to transform their metal cores into corrosion products, even when they are stored in stable climatic conditions [4]. The presence of chlorides raises serious problems about the protection of archaeological artifacts recovered from marine environments. Currently, objects are treated to remove chloride ions using alkaline solutions. The purpose of this operation is to ensure dechlorination on the one hand, and on the other hand to slow down the corrosion rate of metal materials. These treatments have proven their efficiency in most cases, but it still is difficult to get a clear indicator that an artifact shows any corrosion recovery after treatment [5]. The cycles of these treatments are generally several months or several years, based on the volume and corrosion state of the metal artifacts. Therefore, protection methods should be regularly tested and optimized according to the correlation between the existing corrosion products and their metal cores. Analysis is conducive to the efficient development of conservation methods.

The complex properties of archaeological materials make every step of the protection operation a meticulous one. Meanwhile, the rationale for a preservation operation should only be designed and assessed after a complete characterization of the corroded archaeological metal. Metal artifacts unearthed from marine environments have been immersed in a high-chlorine electrolytic environment for hundreds of years. The corrosion state is significantly different in the structure of the rust layers compared to that of metal monuments from an outdoor dry-wet cycle [6]. Few data are dedicated to identifying marine corrosion rust at the microscopic scale, especially for archaeological artifacts [7,8,9]. Researchers believed that the distribution of different components in the rust layer had remarkable effects on future corrosion. In marine environments, the long-term immersion of iron is prone to thick rust forming, with many different phases: oxides, oxyhydroxides, sulphides, sulphates, chlorides, etc. Furthermore, the prior phases of rusts are usually a complex mixture of goethite (α-FeOOH), akaganeite (β-FeOOH), lepidocrocite (γ-FeOOH) and magnetite (Fe_3_O_4_) [10]. Recent articles have shown that the formation of akaganeite requires the simultaneous presence of high concentrations of dissolved Fe(II) and chloride [11]. This condition is extremely common in the sea. Therefore, the existence of akaganeite can directly lead to a variety of localized corrosion phenomena. The rust composition and distribution in archaeological irons are always more complicated than simulations of them in the laboratory, especially under natural marine corrosion conditions of more than 800 years. Determining the state of rust is beneficial to infer its corrosion mechanism and guide the protection of archaeological iron for conservationists.

This article reported on the metallic phase of iron materials from the Southern Song Dynasty of China, and analyzed their rusted state from marine corrosion over hundreds of years, in order to provide useful information for preventing further degradation of iron artifacts after salvation, and ensuring their preservation. These results also contribute to a better understanding of archaeologically complex corrosion systems, which will lead to improved diagnosis and conservation of archaeological and cultural heritage pieces. Therefore, this paper applies multi-scaled, comprehensive methods to study the corrosion of archaeological iron extracted from marine environments. Iron fragments covered with rust from the Nanhai I ship were used for analysis in this research. The rust was mainly analyzed using optical stereo microscopy, micro-Raman spectroscopy and electrochemical impedance spectroscopy, combined with SEM, XRD and FTIR, to evaluate current corrosion characteristics and to find the corrosion causes during the marine period to predict the future corrosion situation for conservationists.

## 2. Materials and Methods

### 2.1. Archaeological Iron Objects

**Figure 1:**
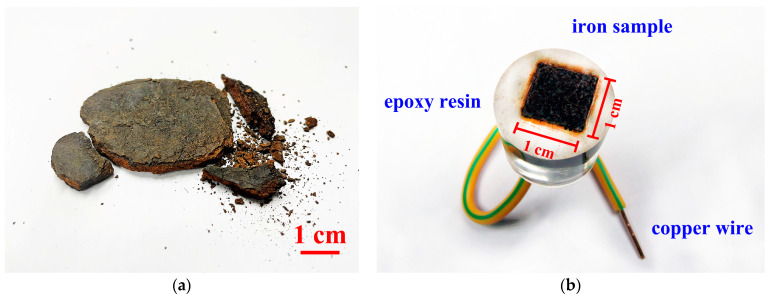
Archaeological iron artifacts from the Nanhai I (**a**); working electrode (**b**).

The iron samples were collected from the Maritime Silk Road Museum of Guangdong. The objects were all completely covered with rust and mud after being salvaged from a marine environment; the silt was gradually removed by spraying. The samples had been dechlorinated in pH = 9–10 sodium hydroxide solution for about 10 years in the archaeological laboratory. The water quality is regularly updated every month by adding about 4 g/t sodium hydroxide to a new soaking tank, and then transferring the iron objects into it. Before this experiment, the concentrations of Cl^−^, NO_3_^−^ and SO_4_^2−^ were determined using ion chromatography to be about 26.93, 4.17 and 4.45 mg/L, respectively. During pretreatment cleaning with deionized water, the rust layer was gradually exposed. The surface appearance and approximate dimensions are shown in Figure 1a.

### 2.2. Sample Preparation

Rust powder: a razor and brush were used to scrape the rust powder from the iron, then an agate mortar and pestle was used to grind the rust to a uniform and fine size. The rust was previously powdered and screened to a particle size of less than 125 µm.

Cross-section of iron core and rust layers: the cross-section was cut from an edge of the archaeological iron and then dried at room temperature for 3 days. The object was embedded in epoxy resin for mechanical grinding with SiC paper (grade 180–2000) under absolute ethanol. The sample was polished with diamond paste, in order to obtain a smooth cross-section.

Working electrode: the electrode was cut from along the above cutting edge, in order to ensure the continuity and similarity of the rust layers and the iron core. The sample was cut into a small piece of 10 mm × 10 mm, and embedded in epoxy resin to protect the cut edges for the electrochemical impedance test. The details are shown in Figure 1b.

### 2.3. Morphology Observation

An optical stereo microscope (Eclipse LV100ND, Nikon, Tokyo, Japan) equipped with a digital camera (DS-Ri2, Nikon, Tokyo, Japan) was used to observe the metallographic structure of the iron core and the appearance of the rust layers.

A scanning electron microscope (Quanta 200F, FEI Company, Hillsboro, OR, USA) was used to show the morphology of the sample surface in detail under an accelerating voltage of 10 kV.

### 2.4. Determination of Rust Composition

X-ray diffraction was recorded by a diffractometer (X’pert-3 Power, PANanalytical, Almelo, The Netherlands) with a Cu anode, in order to determine the crystalline structure of the rust. The generator voltage used was 40 kV, while the tube current was 40 mA. Angular scanning was performed from 5° to 80°, with a scan step size of 0.013°. 

FTIR transmittance analysis (Tensor 27, Bruker, Karlsruhe, Germany) was prepared by KBr-matrix pellets to define chemical structures of rust in the ranges from 400 to 4000 cm^−1^ at 4 cm^−1^ resolution. A powder sample of 0.2 mg was mixed with 200 mg of dry KBr (>99% FTIR grade, Sigma-Aldrich, Burlington, MA, USA), in order to be milled and pressed into pellets.

Micro-Raman spectroscopy was performed on the cross-sectional sample using a micro-Raman spectroscope (Thermo Scientific DXRxi, Thermo Fisher Scientific, Waltham, MA, USA) that was equipped with a microscope (OLYMPUS BX51, Olympus Corporation, Tokyo, Japan) to define different rust components. Raman excitation was provided by a frequency-doubled Nd:YAG laser operating at 532 nm, with a power of about 0.2 mW and with a probe diameter of about 1 mm. All spectra were calibrated using the 519.5 cm^−1^ line of a silicon wafer.

### 2.5. Electrochemical Impedance

Electrochemical impedance spectroscopy of rusted and naked archaeological iron objects was conducted using an electrochemical workstation (CS-350, CorrTestTM, Wuhan, China). Measurements were carried out in a three-electrode cell with 1.5 g/L NaCl and 1.5 g/L Na_2_SO_4_ mixed together to form an electrolyte to simulate sea water. The three-electrode cell included a saturated calomel reference electrode filled with saturated KCl solution which served as a reference electrode; a platinum auxiliary electrode with an exposure surface of 10 mm × 10 mm served as a counter electrode; and the iron objects with an exposure surface of 1 cm^2^ functioned as working electrodes, as described above. Before testing, the electrodes were kept in the solution for 30 min to stabilize the free corrosion potential. An open-circuit potential was applied with frequencies ranging from 100 kHz to 10 mHz, and a sinusoidal perturbation signal with a 10-millivolt amplitude was used. The obtained data were interpreted based on an equivalent circuit using Zview (Zview2, Scribner Associates Inc., Southern Pines, NC, USA), in order to obtain the fitting parameters.

## 3. Results and Discussion

### 3.1. Cracks in the Archaeological Iron

**Figure 2:**
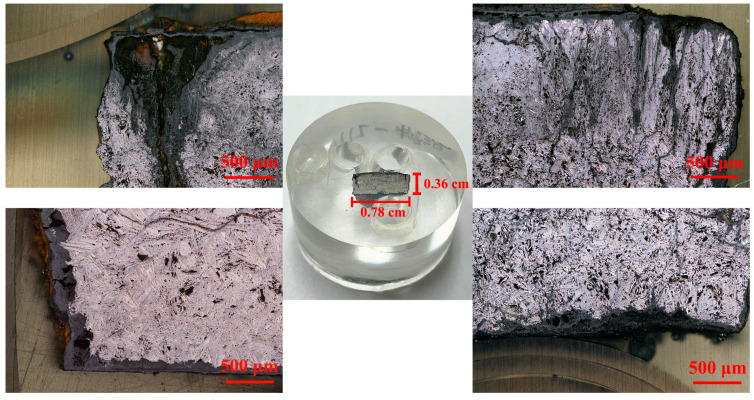
Digital and microscopic graphs of the cross-section of archaeological iron.

After grinding and polishing, the cross-sectional sample in Figure 2 shows a significant corrosion state without any corrosive agent. The cross-section had a length of 0.78 cm and a width of 0.36 cm, which was approximately a rectangular plane. General corrosion and long cracks extending from the rust layer to the iron core were clearly observed, and the corroded state of the upper surface was most obvious. There was a worryingly large crack in the upper left corner, which may be caused by the combined effect of general corrosion and crevice corrosion. Due to the internal stresses of corrosion products [12], the core gradually expanded and resulted in the peeling off of small parts from the metal object. The cracks in the upper right corner were densely distributed and intersecting, extending in different directions to the core; these conditions are not conducive to stable storage of the archaeological iron. This embedded sample would continue to be used for the analysis of the cast state of the iron core and for the composition of the rust layer on its upper surface.

### 3.2. Metallographic Studies of the Archaeological Iron

**Figure 3:**
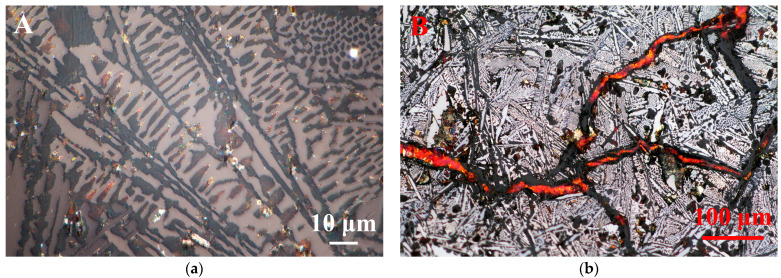
Metallographic diagrams of an archaeological iron artifact from the Nanhai I. Primary cementite and eutectic ledeburite (**a**); cracks of the iron (**b**).

In Figure 3, the silvery white strips in the cross section are primary cementite, and the matrix is eutectic ledeburite. The archaeological sample was a typical hypereutectic white cast iron with high carbon content in the range of 4.3–6.69 wt.%. The elemental analysis was further tested by EDS, and the composition was 5.31% C, 0.11% Si, 0.15% Mn, 0.08% P, 0.05% S and 94.3% Fe, which agreed with the metallographic data.

When the hypereutectic molten iron began to cool, the long and coarse primary cementite was produced. It could grow freely in the liquid, thus it became shaped as strips or flakes. Gradually, the temperature of the metal liquid continued to drop, and a eutectic ledeburite phase formed. Due to local cooling, primary cementite and eutectic ledeburite also arranged in different cooling directions. Generally, two major phases of eutectics were encountered in white cast iron with high carbon content under different undercoolings. They could be described as ledeburite eutectic which had dendrite branches, while another phase was plate-like eutectic. Besides, plate-like eutectic in white cast iron became the main constituent of the microstructure with an increased degree of melt undercooling (ΔT~35–40 °C), while ledeburite eutectic often appeared at lower undercooling (ΔT~5–25 °C). This transformation does not really depend on the starting alloy’s composition (hypo- or hyper-eutectic) [13]. The ledeburite in the metallographic images of the archaeological iron section was mostly dendritic. Based on this, it could be inferred that the iron object from the Southern Song Dynasty experienced slow cooling after being cast into a mold.

### 3.3. Composition of the Rust Powder

**Figure 4:**
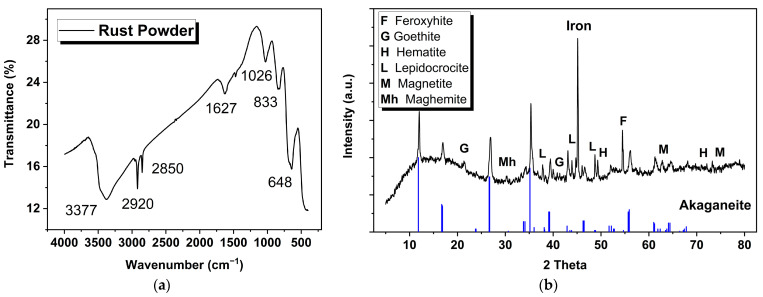
FTIR (**a**) and XRD (**b**) diagrams of rust powder.

The basic components of the rust layer were identified by FTIR. Figure 4 shows that the main components of the powdered rust were akaganeite (β-FeOOH) and lepidocrocite (γ-FeOOH), with a small amount of maghemite (γ-Fe_2_O_3_). The absorption peaks were at 833 cm^−1^ and 1627 cm^−1^, corresponding to the bending of O-H bonds and vibrations of Fe-O bonds in β-FeOOH [14]. A peak at 1026 cm^−1^ was attributed to O-H bending in γ-FeOOH [15]. γ-Fe_2_O_3_ and β-FeOOH overlapped at the characteristic peak of 648 cm^−1^ [16]. As for the peak at 3377 cm^−1^, it represented a common peak from stretching vibrations of the −OH bond [14].

An XRD was used to analyze the composition of rust powder, combined with data from infrared spectroscopy. The XRD spectrum in Figure 4b was compared individually using Jade software (Jade 9.0, MDI, Livermore, CA, USA) and PDF-card references (α-FeOOH: #00-029-0713; β-FeOOH: #00-042-1315; γ-FeOOH: #00-008-0098; δ-FeOOH: #00-013-0087; α-Fe_2_O_3_: #00-001-1053; γ-Fe_2_O_3_: #00-039-1346; Fe_3_O_4_: #00-019-0629). The peaks of the XRD data and the provided standard spectrum of akaganeite clearly corresponded to each other, which may be regarded as the main phase of rust powder. Meanwhile, a significant signal of pure iron appeared, proving that there were some tiny iron particles that peeled off with rust due to corrosion. The peak intensities of other rust components were weak, but a small amount of goethite (α-FeOOH), lepidocrocite, feroxyhite (δ-FeOOH), hematite (α-Fe_2_O_3_), maghemite and magnetite could be confirmed according to the PDF information.

Ferric oxyhydroxides have four common types; goethite is an electrochemically stable phase, whereas others are all active phases that may be transformed into relatively stable magnetite [9]. The results of FTIR and XRD analyses indicated that a large amount of β-FeOOH was present in the rust, which was associated with the high concentration of chloride in the South China Sea. However, in this work, α-FeOOH was only detected in the XRD with a weak intensity, and with a scarce signal in the IR spectra. Most iron-based compounds involved in corrosion reactions are prone to transforming into β-FeOOH and γ-FeOOH in marine environments, resulting in small amounts of α-FeOOH in rust. Generally, formation of β-FeOOH requires chloride ions in order to stabilize the structure of its crystal [17]. Moreover, β-FeOOH is the most detrimental for continuous corrosion of archaeological iron among all the ferric oxyhydroxides [18]. Therefore, the composition of the rust powder preliminarily indicated that the rust phase of this archaeological iron had not yet reached stability, and that the subsequent stabilization treatment was indeed required.

### 3.4. Structure of the Rust Layers

**Figure 5:**
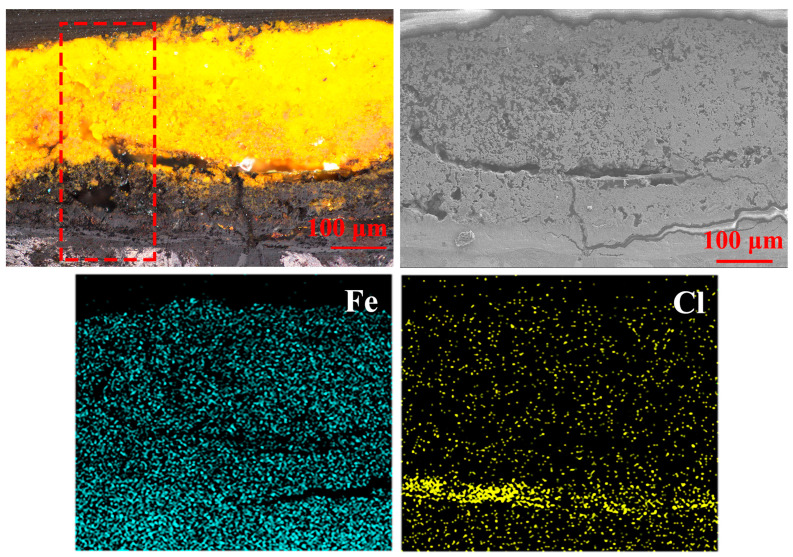
Graphs of the microstructure and element distribution of rust layer A (red square for Raman analysis).

Figure 5 shows details of rust layer A, which is marked in Figure 2. The surface rust of archaeological iron had an obvious dividing line, separating two layers: a yellow outer layer that was mostly loose and porous, and an inner layer near the iron core that was almost dominated by black dense rust. Large cracks ran through the entire rust to the iron core, such that the rust layer could not completely protect the internal metal, and tended to peel off. The iron element was almost evenly distributed throughout the rust. The chlorine element was evenly distributed in the outer rust, and formed a highlight band that coincided with the position of the dense rust layer.

**Figure 6:**
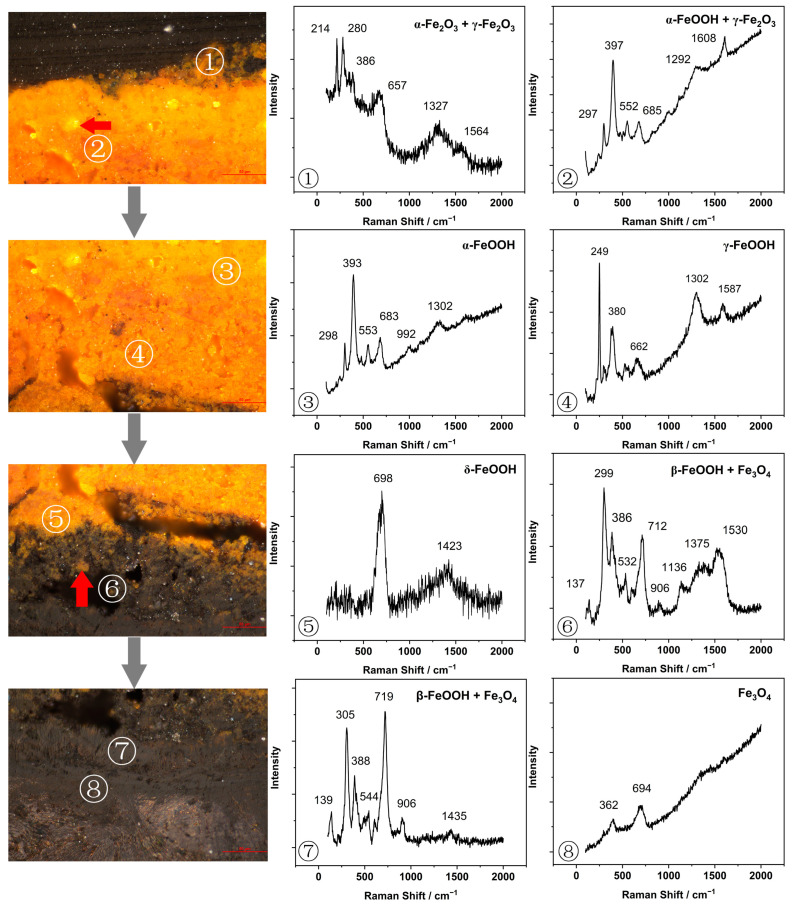
Raman spectra of rust layer A (red square in Figure 5). ①–⑧ were the main points for Raman analysis.

Micro-Raman analysis revealed eight points, as shown in Figure 6. The oxides and hydroxides of iron compounds were identified, layer by layer, according to the literature’s values [10,19,20]. The cross-section of rust layer A indicated four distinct layers based on color: a yellow outermost layer dotted with light-yellow spots (I); a yellow layer (II); a black layer dotted with yellow spots (III); and a black innermost layer which was closely connected with the iron core (IV). Raman spectra showed that the outermost rust was mainly formed by hematite (α-Fe_2_O_3_) and maghemite (γ-Fe_2_O_3_), with characteristic peaks at 214, 280 and 386 cm^−1^ to α-Fe_2_O_3_, and a relatively weak peak at 1608 cm^−1^ to γ-Fe_2_O_3_. The light-yellow dots in the outer rust showed a strong peak at 393 cm^−1^, with less intense peaks at 298, 683 and 1302 cm^−1^ that were attributed to the formation of goethite (α-FeOOH). The strongest peak was close to 249 cm^−1^, and a second peak in the Raman shift of 380 cm^−1^ was related to the lepidocrocite phase (γ-FeOOH) that coexisted with α-FeOOH phase. The presence of feroxyhite (δ-FeOOH) was confirmed by its characteristic bands at a single peak of 698 cm^−1^ at the junction between the black and yellow rust. Akaganeite (β-FeOOH), whether in marine environments or soils, is usually hygroscopic and is easily converted into other oxides, especially magnetite (Fe_3_O_4_) [21]. Furthermore, chloride ions are often present in the lattice of β-FeOOH, which can lead to a complete loss of artifacts [10]. In this particular object, akaganeite had bands at 137, 299 and 386 cm^−1^, while magnetite was assigned the Raman bands at 362 and 694 cm^−1^. The two kinds of rust had obvious signals near the black dense layer, where chlorine was enriched.

**Figure 7:**
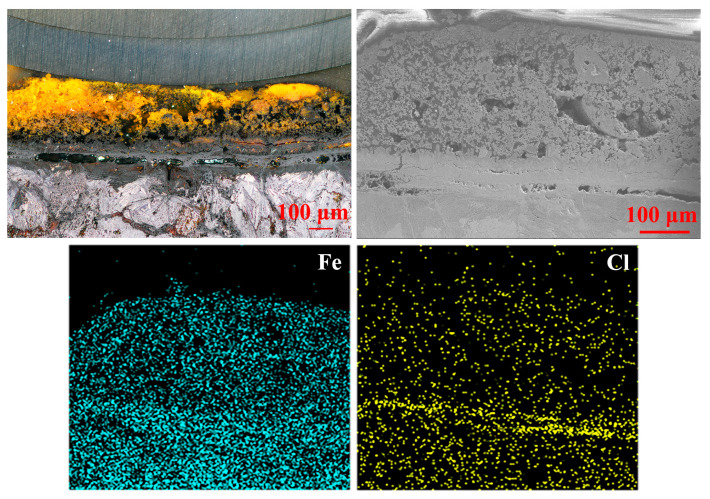
Graphs of the microstructure and element distribution of rust layer B.

**Figure 8:**
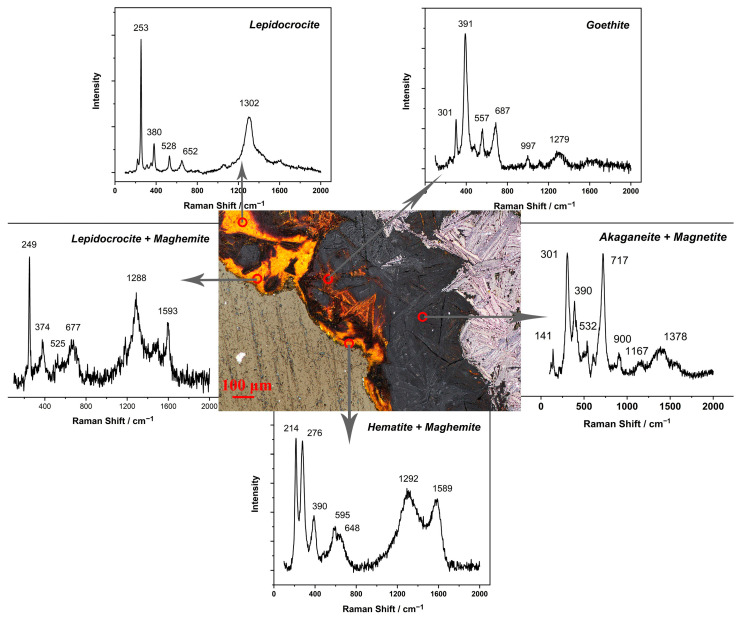
Raman spectra of rust layer B.

Area B in Figure 7 and Figure 8 was the rust layer on the upper right of the iron sample. There were several wide and hollow lines at the interface between rust and iron, which did not tightly wrap around the internal metal to protect it against the external environment. The overall structure was similar to that of layer A, except that the black rust gradually occupied the yellow outer layer. Maghemite, goethite and lepidocrocite were the dominant phases in the yellow loose rust layer, and the black dense layer where chlorine gathered was still mainly composed of akaganeite and magnetite.

Therefore, there were two obvious rust layers on the upper surface of the iron. The loose yellow rust had a tendency to exfoliate, and the dense black rust had a lot of cracks, hence the inner iron core still had channels that were in contact with the external environment, making it difficult for the material to remain stable.

### 3.5. Corrosion Evaluation in Simulated Seawater

Electrochemical impedance spectroscopy (EIS) is an effective analysis tool that can be used to obtain valuable information about the nature of the rust layers formed on the marine-corroded archaeological iron.

**Figure 9:**
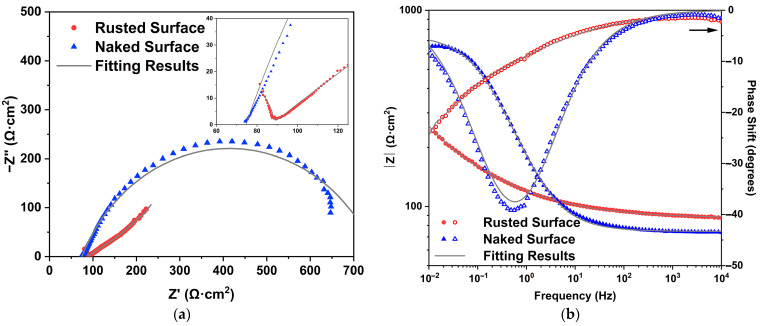
Nyquist (**a**) and Bode (**b**) plots of naked and rusted archaeological iron in simulated seawater.

**Figure 10:**
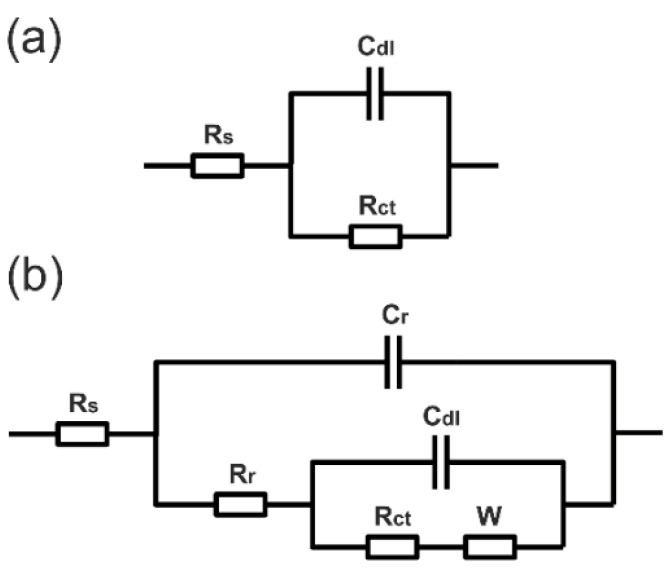
Equivalent circuits of naked (**a**) and rusted (**b**) archaeological iron in simulated seawater.

Figure 9 shows the Nyquist and Bode plots of naked (removing the rust) and rusted archaeological iron, respectively. For the rusted surface, the Nyquist plot is composed of a depressed capacitive semi-arc in the high frequency range, and a long tail in the low frequency region that represents typical Warburg impedance. This indicates that the electrochemical corrosion process on the rust/iron interface in the NaCl/Na_2_SO_4_ solution was mainly controlled by the diffusion process. The charge transfer resistance of the iron rust dominates at the medium-low frequency region, while the rust resistance dominates at the high frequency region [22]. The construction of rust layers could be described using the equivalent circuit in Figure 10b. After the rust surface was completely removed, the archaeological sample reacted with the simulated seawater directly. Only one double-layer capacitive semicircle was observed in the Nyquist plot, and its equivalent circuit is shown in Figure 10a. R_s_ represents the electrolyte resistance, C_r_ the rust capacitance, R_r_ the rust resistance, C_dl_ the double-layer capacitance, R_ct_ the charge transfer resistance, and W the Warburg resistance or barrier diffusion impedance. Diffusion impedance was regarded as the diffusion of corrosive electrolyte through pores in the rust layer that acted as a diffusion barrier. The constant phase angle element (CPE) was introduced to describe the C_dl_ and C_r_ in the fitting circuits due to the rough and uneven surfaces of the sample [23].

The total impedance for the circuit in Figure 10a could be expressed by the following equation:

(1) $${Z=R}_{s}+\frac{1}{Y_{\mathrm{dl}}{\left(j\omega \right)}^{n_{\mathrm{dl}}}+\frac{1}{R_{\mathrm{ct}}}}$$

The electrochemical impedance for the circuit in Figure 10b could be expressed by the following equation:

(2) $${Z=R}_{s}+\frac{1}{Y_{r}{\left(j\omega \right)}^{n_{r}}+\frac{1}{R_{r}{+1/[Y}_{\mathrm{dl}}{\left(j\omega \right)}^{n_{\mathrm{dl}}}{+1/(R}_{\mathrm{ct}}{+Z}_{W})]}}$$

where Y_r_ and n_r_, and Y_dl_ and n_dl_ are constants representing the elements C_r_ and C_dl_, respectively. Z_w_ is the Warburg resistance, represented as follows [18]:

(3) $$Z_{W}{=A}_{W}{\cdot (j\omega )}^{-0.5}$$

where A_W_ is the modulus of Z_W_.

The equivalent circuits of Figure 10a,b were used to fit the Nyquist and Bode plots of rusted and naked iron, respectively. Fitting data are listed in Table 1. R_ct_ of the rusted iron was 17.01 Ω·cm^2^, and was much smaller than that of the naked iron; this was attributed to the reduction of the rust that accelerated the cathodic reaction. The lower R_ct_ value indicated that the corrosion products formed on the archaeological object were less compact, less continuous and porous [22]. R_r_ is usually used to evaluate the corrosion resistance of rust [17]. Therefore, it could be that the rust layer has poor barrier behavior, and can not effectively prevent the corrosion of the iron core, which is consistent with the porous structure of the rust layer mentioned previously.

### 3.6. Mechanism of the Rust Growth

The rust growth and iron corrosion mechanism of this archaeological iron could be proposed from the iron surface to the outer rust layer. Since the rust growth of archaeological iron was not an independent process of a single rust phase, it was better to describe the rust growth according to environmental changes over multiple periods.

When the Nanhai I became shipwrecked, the cargo on the ship sank into the sea. The iron products became immersed in a salt-rich water environment and began corroding. The corrosion reactions during this initial stage could have occurred as follows [24]:

The iron objects functioned as an anode region:

(4) $$\mathrm{Fe}\;\to {\;\mathrm{Fe}}^{2+}{+2e}^{-}$$

As the ship sank, a large amount of air would have been brought in instantly, and attached to the surface of the cargo in the form of bubbles. Hence, the interface between iron and sea water would be a cathodic reduction due to the oxygen dissolved in the thin water film. Meanwhile, countless electrochemical cells involving cathodic and anodic areas began reacting, and scattered on the iron surface to form numerous corrosion sites:

(5) $$O_{2}{+2H}_{2}{O+4e}^{-}\;\to {\;4\mathrm{OH}}^{-}$$

Then, the interface would quickly turn into normal seawater with a high chloride concentration, with the consumption and bursting of tiny bubbles. The chloride ions were more readily adsorbed than oxygen in competition for surface sites, and consequently became deposited on the metal with high retention, promoting the formation of ferrous chloride and accelerating the corrosion of iron. Moreover, the accompanying hydrolysis reaction began to create a weakly acidic environment around it [25]:

(6) $${\mathrm{Fe}}^{2+}{+2\mathrm{Cl}}^{-}\;\to {\;\mathrm{FeCl}}_{2}$$

(7) $${\mathrm{FeCl}}_{2\;}{+2H}_{2}O\;\to \;\mathrm{Fe}{\left(\mathrm{OH}\right)}_{2}{+2\mathrm{Cl}}^{-}{+2H}^{+}$$

Mud and sand would gradually envelop and cover the archaeological objects with surrounding water flow, thereby blocking the update and circulation of dissolved oxygen and reserving the chloride ions around the iron. The high chloride concentration and local acidic conditions at the interface increased the chance formation of FeOOH, especially akaganeite and lepidocrocite [15]:

(8) $$4\mathrm{Fe}{\left(\mathrm{OH}\right)}_{2}{+O}_{2}\to {4\beta ,\gamma -\mathrm{FeOOH}+2H}_{2}O$$

Meanwhile, some ferrous ions were not chosen to become compounds with chloride ions, instead forming hydrated ions in solution or becoming oxidized to Fe(OH)_3_, which has very low solubility. The intermediate corrosion products FeOH^+^ and Fe(OH)_3_ could be converted into γ-FeOOH and δ-FeOOH, with small amounts of oxygen remaining in the mud and sand [24]:

(9) $${\mathrm{Fe}}^{2+}{+H}_{2}O\;\to {\;\mathrm{FeOH}}^{+}{+H}^{+}$$

(10) $${2\mathrm{FeOH}}^{+}{+O}_{2}{+2e}^{-}\;\to \;2\gamma -\mathrm{FeOOH}$$

(11) $$2\mathrm{Fe}{\left(\mathrm{OH}\right)}_{2}{+O}_{2}{+H}_{2}O\;\to \;2\mathrm{Fe}{\left(\mathrm{OH}\right)}_{3}$$

(12) $$\mathrm{Fe}{\left(\mathrm{OH}\right)}_{3}\;\to {\;\gamma ,\delta -\mathrm{FeOOH}+H}_{2}O$$

Eventually, the archaeological iron became wrapped and buried tightly under the silt in deep sea, forming an anaerobic and weakly acidic environment. Fe_3_O_4_ could be reduced and converted rapidly from γ-FeOOH and β-FeOOH [26]. Ferrous ions and electrons could pass through an Fe_3_O_4_ layer due to its good conductivity. Moreover, Fe_3_O_4_ would have been prone to accumulate at the interface and form a dense layer on the surface of iron under the cathodic reaction. The reduction of the γ-FeOOH and β-FeOOH layer would continue its transformation into the cathode area of the Fe_3_O_4_ layer. Meanwhile, the chloride ions would gradually cause local breaks in the oxide/oxyhydroxide film on the iron objects, especially at rough and uneven areas, thereby making the corrosion process of the archaeological iron slow but unstoppable in its marine environment for more than 800 years:

(13) $${3\beta ,\gamma -\mathrm{FeOOH}+H}^{+}{+e}^{-}\;\to {\;\mathrm{Fe}}_{3}O_{4}{+2H}_{2}O$$

(14) $${8\beta ,\gamma -\mathrm{FeOOH}+\mathrm{Fe}}^{2+}{+2e}^{-}\;\to {\;3\mathrm{Fe}}_{3}O_{4}{+4H}_{2}O$$

After being excavated out of seawater, the archaeological iron was immersed in a weakly alkaline solution and then transported to the laboratory for research. For preservation and display, this iron object was placed at room temperature in order to gradually remove internal moisture. Since the oxygen concentration and humidity changed significantly on the rust surface during the drying stage, it became prone to forming γ-FeOOH again as well as α-FeOOH. At this stage, γ-FeOOH and α-FeOOH could be generated by oxidizing the precipitation of Fe(OH)_2_ derived from the outer rust, and only a fraction of FeOOH was generally adsorbed on the surface of the outermost layer. The structure of newly generated FeOOH was loose and did not cover the object tightly, resulting in the formation of pores in yellow rust. Hence, when the archaeological iron was removed from its dechlorination solution or rinsed with distilled water, it became easily detached. After immersion in alkaline solution, FeOOH in the dehydration stage was more likely to form maghemite and hematite [21]:

(15) $$4\mathrm{Fe}{\left(\mathrm{OH}\right)}_{2\;}{+O}_{2}\;\to {\;4\alpha ,\gamma -\mathrm{FeOOH}+2H}_{2}O$$

(16) $$\mathrm{Fe}{\left(\mathrm{OH}\right)}_{3}\;\to {\;\alpha ,\gamma -\mathrm{FeOOH}+H}_{2}O$$

(17) $$2\alpha ,\gamma -\mathrm{FeOOH}\;\to {\;\mathrm{Fe}}_{2}O_{3}\cdot H_{2}O\;\to {\;\mathrm{Fe}}_{2}O_{3}{+H}_{2}O$$

## 4. Conclusions

Archaeological iron artifacts excavated from the Nanhai I ancient ship were corroded and fragmented on a large scale. The metallographic structures of these objects belonged to hypereutectic white cast iron with a carbon content of 4.3–6.69 wt.%, and experienced low-melt undercooling. There were many cracks in the iron core that were caused by general corrosion, which was the direct cause of the fragmentation.

Furthermore, the upper rust of the archaeological iron that was most corroded could be distinguished as two layers. The outer layer was loose yellow rust mainly composed of α-Fe_2_O_3_, γ-Fe_2_O_3_, α-FeOOH, γ-FeOOH and δ-FeOOH. The dense, black rust layer close to the iron core was gathered β-FeOOH and Fe_3_O_4_, full of chlorine that threatened to break this dense layer. Meanwhile, there were also many cracks in the rust layers that extended to the iron surface, resulting in a very low resistance against the seawater. This indicated that the rust was a poor barrier for the internal metal. These two features were the important reasons behind the observed general corrosion of the archaeological iron.

There was a reasonable mechanism proposed to explain the growth and transformation of each rust layer, as well as to explain the reason why the rust layer cracked and lost its protective effect; the mechanism combines corrosion conditions and reactions from the initial stage of being submerged in seawater with the condition of the artifacts before laboratory protection.

In view of the desalination treatment that had been applied for ten years, it was not recommended to maintain a single desalination operation. For better conservation of these archaeological iron pieces, they should be properly stabilized and protected using corrosion inhibition and rust transformation for iron oxyhydroxides. Moreover, the preservation status should be monitored through a daily routine. These results also contribute to a better understanding of archaeologically complex corrosion systems, which may lead to improved diagnoses and conservation of archaeological and cultural heritage pieces by conservationists and archaeologists.

## Figures and Tables

**Table 1:** Fitting data of impedance parameters in equivalent circuits.

Samples	R_s_ (Ω·cm^2^)	Y_dl_ (mF·cm^−2^·s^−*n*^)	*n*_dl_	R_ct_ (Ω·cm^2^)	R_r_ (Ω·cm^2^)	Y_r_ (μF·cm^−2^·s^−*n*^)	*n*_r_	A_W_ (Ω·cm^2^·s^−0.5^)	χ^2^
Naked	75.28	1.80	0.74	675.70					0.056
Rusted	42.10	8.32	0.31	17.01	42.65	0.012	0.92	0.41	0.011

## Data Availability

The data presented in this study are available in the article.
